# How satisfied are cervical dystonia patients after 3 years of botulinum toxin type A treatment? Results from a prospective, long-term observational study

**DOI:** 10.1007/s00415-019-09527-2

**Published:** 2019-09-09

**Authors:** Carlo Colosimo, David Charles, Vijay P. Misra, Pascal Maisonobe, Savary Om, the INTEREST IN CD2 study group, A. Abdulnayef, N. U. Adatepe, M. A. Araujo Leite, S. Badarny, O. Bajenaru, M. Bares, P. Bejjani, B. Bergmans, R. Bhidayasiri, H. Bozic, F. E. Cardoso Costa, C. Carlstrom, G. Castelnovo, M. H. Chang, Y. Y. Chang, T. M. Chung, M. Coletti-Moja, V. Delvaux, P. Dioszhegy, O. Dogu, W. Duzynski, E. Ehler, L. Espinosa Sierra, G. Fabbrini, J. Ferreira, A. Ferreira Valadas, C. Foresti, P. Girlanda, K. J. Goh, A. Graca Velon, S. Grill, T. Gurevitch, M. Hadidi, M. A. Hamimed, A. Hamri, T. Harrower, S. Hassin, P. Hedera, J. F. J. G. Hernandez, J. Hernandez Franco, B. Ho, S. L. Ho, A. Hughes, T. Ilic, J. S. Inshasi, C. W. Ip, S. Jamieson, R. D. G. Jamora, R. Jech, B. S. Jeon, A. Kaminska, M. Karpova, D. Khasanova, J. M. Kim, J. W. Kim, C. Y. Kok, A. Korenko, J. Korv, S. Koussa, T. Kovacs, A. Kreisler, P. Krystkowiak, W. Kumthornthip, C. H. Lin, F. Lundin, G. Lus, M. Magalhaes, A. N. Masmoudi, R. Mercelis, A. Misbahuddin, C. Moebius, B. Mohammadi, B. Nazem, K. Ng, G. Nurlu, J. Nyberg, D. Nyholm, S. Ochudlo, P. Otruba, R. Pfister, Z. Pirtosek, D. Pokhabov, S. Quinones Aguilar, G. Quinones Canales, S. Raghev, H. Rickmann, M. Romano, R. L. Rosales, I. Rubanovits, V. Santilli, L. Schoels, M. Simonetta-Moreau, M. A. Simu, Y. H. Sohn, S. Soulayrol, I. Supe, M. Svetel, T. Sycha, E. K. Tan, S. Timerbaeva, A. B. Tokcaer, R. Trosch, V. Tugnoli, V. Tumas, C. Van der Linden, A. Vetra, C. Vial, E. Vidry, D. Williams, S. Wimalaratna, C. Yiannikas

**Affiliations:** 1 Department of Neurology, Santa Maria University Hospital, Viale Tristano di Joannuccio 1, 05100 Terni, Italy; 2 Department of Neurology, Vanderbilt University Medical Center, Nashville, TN USA; 3 Department of Neurology, Imperial College Healthcare NHS Trust, London, UK; 4 Ipsen Pharma, Boulogne-Billancourt, France

**Keywords:** Botulinum toxin, Cervical dystonia, Observational study, Satisfaction, Treatment

## Abstract

**Background:**

Patients with cervical dystonia (CD) typically require regular injections of botulinum toxin to maintain symptomatic control. We aimed to document long-term patient satisfaction with CD symptom control in a large cohort of patients treated in routine practice.

**Methods:**

This was a prospective, international, observational study (NCT01753349) following the course of adult CD treated with botulinum neurotoxin type A (BoNT-A) over 3 years. A comprehensive clinical assessment status was performed at each injection visit and subjects reported satisfaction in two ways: satisfaction with symptom control at peak effect and at the end of treatment cycle.

**Results:**

Subject satisfaction remained relatively stable from the first to the last injection visit. At 3 years, 89.9% of subjects reported satisfaction with symptom control at peak effect and 55.6% reported satisfaction with symptom control at end of treatment cycle. By contrast, objective ratings of CD severity showed an overall reduction over 3 years. Mean ± SD Toronto Western Spasmodic Rating Scale (TWSTRS) Total scores (clinician assessed at end of treatment cycle) decreased from 31.59 ± 13.04 at baseline to 24.49 ± 12.43 at 3 years (mean ± SD reduction from baseline of − 6.97 ± 11.56 points). Tsui scale scores also showed gradual improvement; the percent of subjects with a tremor component score of 4 reduced from 12.4% at baseline to 8.1% at 3 years.

**Conclusions:**

Despite objective clinical improvements over 3 years, subject satisfaction with symptom control remained relatively constant, indicating that factors other than symptom control also play a role in patient satisfaction.

**Electronic supplementary material:**

The online version of this article (10.1007/s00415-019-09527-2) contains supplementary material, which is available to authorized users.

## Introduction

Primary cervical dystonia (CD) is the most common type of adult focal dystonia and is primarily characterised by involuntary twisting or turning of the neck causing an abnormal head position [1–3]. Disability with functional impairment, pain, and embarrassment with social withdrawal are often prominent features of CD and numerous studies highlight the negative impact of CD symptoms on everyday functioning and quality of life [4–7]. Over the past 3 decades, local chemodenervation with botulinum neurotoxin type A (BoNT-A) has become the first-line and mainstay of therapy for people living with CD [8, 9]. Although the evidence levels differ across BoNT serotypes and brands [9], abobotulinumtoxinA (aboBoNT-A), incobotulinumtoxinA (incoBoNT-A), onabotulinumtoxinA (onaBoNT-A) and rimabotulinumtoxinB (rimaBoNT-B) each have regulatory approval for CD across several countries, and are commonly used.

In the pre-BoNT-A era, natural history studies reported an initial worsening of symptoms over the first years before a period of symptom stabilisation [10]. As a rule, primary CD is not associated with spread to other body parts, although a proportion of patients will also have postural limb tremor and/or another focal dystonia [11]. Remission from CD symptoms is rare, and most patients repeatedly return for BoNT-A re-injection to maintain control of their symptoms [12–16]. Since the average age of diagnosis is around 40 years old [17], patients usually need treatment over decades. Surveys of CD patients have found that the most common reason for stopping BoNT-A treatment is a perceived lack of efficacy, even when there is a clear treatment effect on clinical scales [18]. Other studies have also identified the inconvenience of frequent injections (e.g., long travel distances and costs) and high out-of-pocket costs as other possible causes of discontinuation [16, 19]. Such data indicate the need to set reasonable patient expectations of treatment [7].

Patient-reported outcomes (PROs) are increasingly recognized as key measures of effectiveness as they provide ‘real-life’ information about a given intervention from the patient’s perspective [20, 21]. In the case of CD, patient satisfaction is considered especially important because it is known to directly correlate with willingness to continue treatment [15, 16]. The primary aim of this large, long-term, observational study was to document long-term (over 3 years) patient’s satisfaction with CD symptom control and identify prognostic factors for satisfaction in a large cohort.

## Methods

INTEREST IN CD2 (NCT01753349) was a 3-year multicentre longitudinal cohort study following the course of adult idiopathic CD patients treated with BoNT-A. Baseline analyses have previously been reported [22]. The study was conducted in compliance with the International Society for Pharmacoepidemiology (ISPE) Guidelines for Good Pharmacoepidemiology Practices (GPP) [23]; it began on 10 December 2012 and the last visit occurred on 25 September 2017. Independent Ethics Committee/Institutional Review Board approval was obtained prior to each centre initiation. Written informed consent was obtained prior to subject enrolment and performance of any study procedures.

### Population

Specialist centres recruited adult subjects (≥ legal age in each country) with primary CD presenting for treatment with BoNT-A in routine clinical practice. To avoid selection bias, sites were asked to recruit 8 consecutive subjects during BoNT-A consultations during a defined period or 8 subjects recruited according to predefined frequency (e.g., every third patient) if consecutive inclusions were not feasible. Subjects could be new to BoNT-A treatment or previously treated with BoNT, provided there had been at least a 12-week interval between the last injection and study entry. During the study, subjects could be treated with any BoNT-A formulation, but the decision to treat was taken prior to, and independently from, the decision to offer enrolment to the subject for participation in the study.

### Study visits and assessments

There were four visit types in the study: Baseline Visit, Injection Visits, End of Study Visit, and Early Discontinuation Visit. Visits were scheduled according to the Investigator’s usual practice. All subjects underwent a comprehensive clinical CD assessment at every injection visit.

The primary measure of effectiveness was subject satisfaction with symptom control. At every visit, subjects self-reported their satisfaction in two ways:

1. Recall of their highest level of satisfaction at any time since the last BoNT-A injection (i.e., peak of BoNT-A effect, ‘Highest Satisfaction’).
2. Rating of their current level of satisfaction with symptom control (i.e., end of injection cycle, ‘Today Satisfaction’).

Both types of satisfaction were rated on a 5-point Likert scale (1. completely satisfied; 2. rather satisfied; 3. neither satisfied nor dissatisfied; 4. rather dissatisfied; 5. completely dissatisfied).

Clinical outcomes including the Toronto Western Spasmodic Torticollis Rating Scale (TWSTRS) [24] and Tsui scale (tremor component) [25] were also assessed as secondary measures. Data were collected using an electronic case report form (eCRF) with sections for patterns of dystonia (e.g., rotation, laterocollis etc.), injection parameters (muscles selected, injected dose, injected volume, number of injection sites, use of injection guidance technique), TWSTRS, and Tsui tremor scale scores. At the start of the study, a survey was sent to all sites to ascertain whether they reinjected according to fixed re-injection intervals (hospital rules/usual practice), a flexible schedule (depending on patient needs) or a mix of both.

### Safety

No safety assessments were planned in this study; hence no safety data were recorded in the clinical database. However, in compliance with the European Medicines Agency guideline on good pharmacovigilance practices (GVP Module VI), investigators were required to report all serious adverse events (sAEs) and all study drug-related non-serious adverse events (AEs) occurring with aboBoNT-A to the sponsor’s pharmacovigilance department. Safety data for other BoNT-A were to be reported in line with national requirements but were not available for analyses. We made an effort to review all aboBoNT-A safety data reported and cross-checked with this study.

### Statistical analyses

We estimated that a sample size of 1050 subjects would allow to estimate proportions of satisfied/dissatisfied subjects with a precision of at most 3% based on a two-sided 95% confidence interval. Primary analyses of effectiveness were based on the Main Study Population (MSP), which includes all patients treated with BoNT-A at baseline, with ≥ 1 post-baseline satisfaction assessment and ≥ 1 post-baseline TWSTRS assessment. The statistical analyses of this report are primarily descriptive. Mean and standard deviation (mean ± SD) or median measures were used to summarise continuous variables, and absolute and relative frequencies expressed as percentage (%) are presented for categorical information. Satisfaction with symptom control was predefined as a score of 1 or 2 (completely satisfied or rather satisfied). Reported rates of patient satisfaction with symptom control at the baseline visit were for the subgroup of patients who had been previously treated with BoNT and assessed previous BoNT cycle prior to study entry. Satisfaction throughout the study was assessed from Cycle 2 to the last visit.

Multivariate analyses were performed to identify the factors associated with Today and Highest Satisfaction at 3 years. In cases of multiple injections after 1005 days from the baseline injection, the 3-year visit was defined as the injection visit closest to 1098 days from baseline injection. The following 3-step procedure was used:

1. Univariate logistic regression analyses were used to identify factors potentially associated with satisfaction. The dichotomous variable where 1 = Satisfied and 0 = Not satisfied was described and compared according to potential associated factors. All predictors with a *p* value < 0.20 in the univariate analyses were retained for the next step.
2. Each retained parameter was tested with the others at the 0.001 level, to check for presence of any strong association between them. Associations were tested using the Pearson correlation for continuous variables, the Spearman’s correlation coefficient for mixed categorical and continuous variables, *t* tests for mixed binary and continuous variables and Chi-square test for categorical variables. If independence was not shown for two parameters (*p* < 0.001), the factor with most clinical relevance was retained.
3. Finally, a multivariate analysis to select the most relevant model using a stepwise logistic regression model was conducted to identify the prognostic factors for satisfaction (modelling the probability of being satisfied or not). A significance level of 0.2 was required to allow a variable into the model and a significance level of 0.05 was required to retain variables in the model.

## Results

**Fig. 1:**
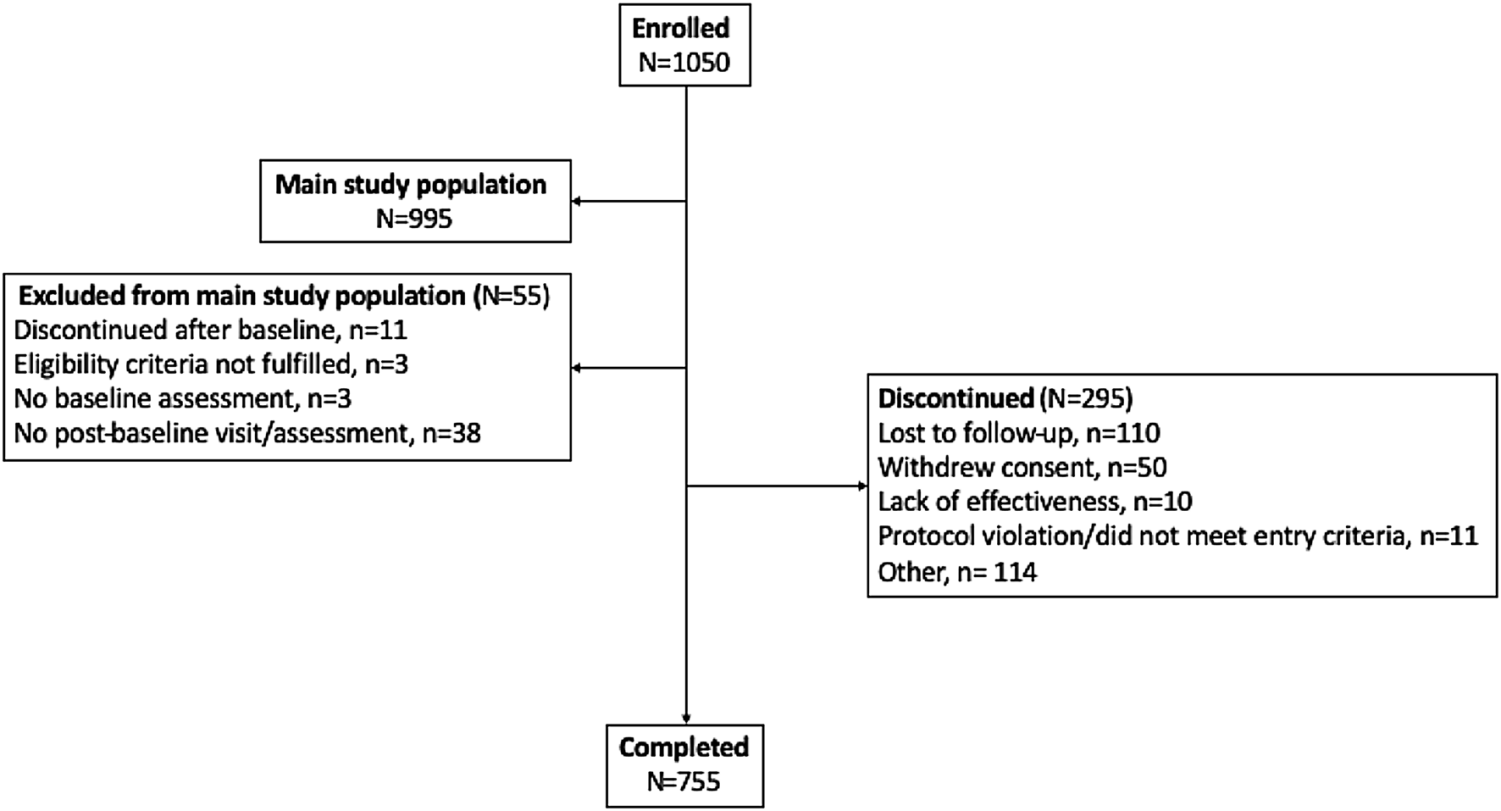
Patient flow through the study

The study included 113 active investigational sites in 34 countries. Of the 1050 enrolled subjects, 995 (94.8%) met criteria for the MSP and 55 (5.2%) subjects were excluded, with the most common reason being that the subject had no post-baseline visit (Fig. 1). Baseline characteristics for the MSP population are presented in Table 1 and were similar to the interim data previously reported [22]. Most subjects were female (68.0% overall) and the mean ± SD age was 54.8 ± 13.1. Most subjects (73.9%) had a complex pattern (i.e., ≥ 2 patterns) at baseline, with rotation (66.2%) and laterocollis (23.0%) being the most common predominant CD patterns.

**Table 1:** Baseline characteristics (main study population)

Demographics	*N* = 995
Age (years); mean ± SD	54.8 ± 13.1
Categories: *n* (%)	
18–30	40 (4.0)
31–40	107 (10.8)
41–50	211 (21.2)
51–60	277 (27.8)
61–70	242 (24.3)
> 70	118 (11.9)
Female/male; *n* (%)	677 (68.0)/ 318 (32.0)
Region; *n* (%)	
Asia	125 (12.6)
Australia	38 (3.8)
Europe	610 (61.3)
Latin America	79 (7.9)
North Africa and Middle East	107 (10.8)
USA	36 (3.6)
CD characteristics	
Type of CD; *n* (%)	
Sporadic	932 (93.7)
Familial	63 (6.3)
Time since diagnosis (years); mean ± SD	8.7 ± 8.1
Categories; *n* (%)	
< 1	72 (7.2)
1–5	361 (36.3)
> 5	562 (56.5)
TWSTRS score; mean (SD)	
Total	31.59 ± 13.04
Severity	15.90 ± 5.66
Disability	9.39 ± 6.28
Pain	6.29 ± 4.86
Predominant CD pattern; *n* (%)	*N* = 993
Rotation	657 (66.2)
Laterocollis	228 (23.0)
Retrocollis	59 (5.9)
Anterocollis	20 (2.0)
Lateral shift of the column	15 (1.5)
Sagittal shift of the column	10 (1.0)
Not applicable*	4 (0.4)
Other head/neck components; *n* (%)	*N* = 993
Shoulder elevation	501 (50.5)
Tremor	486 (48.9)
Jerk	95 (9.6)
Not applicable	234 (23.6)

*CD* cervical dystonia, *SD* standard deviation, *TWSTRS* Toronto Western Spasmodic Torticollis Rating Scale, *USA* United States of America

### Study exposure to BoNT-A treatment

The mean ± SD length of study exposure was 34.20 ± 9.90 months and number of treatment cycles was 8.65 ± 3.25. At baseline, 689 subjects received aboBoNT-A, 247 subjects received onaBoNT-A and 59 subjects received incoBoNT-A. Injection practices were generally in line with BoNT-A prescribing information (Table 2). As previously reported for baseline interim data [22], the most frequently injected muscle groups at baseline/first visit were the splenius capitis, sternocleidomastoid, trapezius, levator scapulae, semispinalis capitis, and scalene group. These six muscle groups remained the most frequently injected muscles throughout the study. Most subjects were reinjected at intervals of 12–16 weeks (58.6%) and switches between BoNT-A preparations were uncommon (14.9%).

**Table 2:** BoNT-A treatment parameters

Injection parameters	*N* = 995
Number of injection cycles; median [range]	10 [1–17]
Dose (units); median [range]	
aboBoNT-A (*N* = 614)	500.0 [50.0–1833.3]
incoBoNT-A (N = 44)	198.6 [45.6–514.3]
onaBoNT-A (*N* = 186)	150.0 [13.3–500.0]
Number of muscles injected; median [range]	4.25 [1.0–18.2]
Injection interval (days)	
Mean ± SD	121.5 ± 48.4
Median [range]	107.0 [44.0 –547.0]
Mean injection interval categories (weeks); *n* (%)	
< 12 weeks	11 (1.1)
12–16 weeks	580 (58.6)
> 16 weeks	399 (40.3)

*aboBoNT-A* abobotulinumtoxinA, *BoNT-A* botulinum neurotoxin type A, *incoBoNT-A* incobotulinumtoxinA, *onaBoNT-A* onabotulinumtoxinA, *SD* standard deviation

Of the 113 active sites, 68 sites treating 583 subjects completed the survey of injection schedule practice. Of these, 7.2% of subjects were injected at fixed re-injection intervals, 37.4% with a flexible schedule (depending on patient needs) and 55.4% with a mix of both.

### Subject self-reported satisfaction with symptom control

**Fig. 2:**
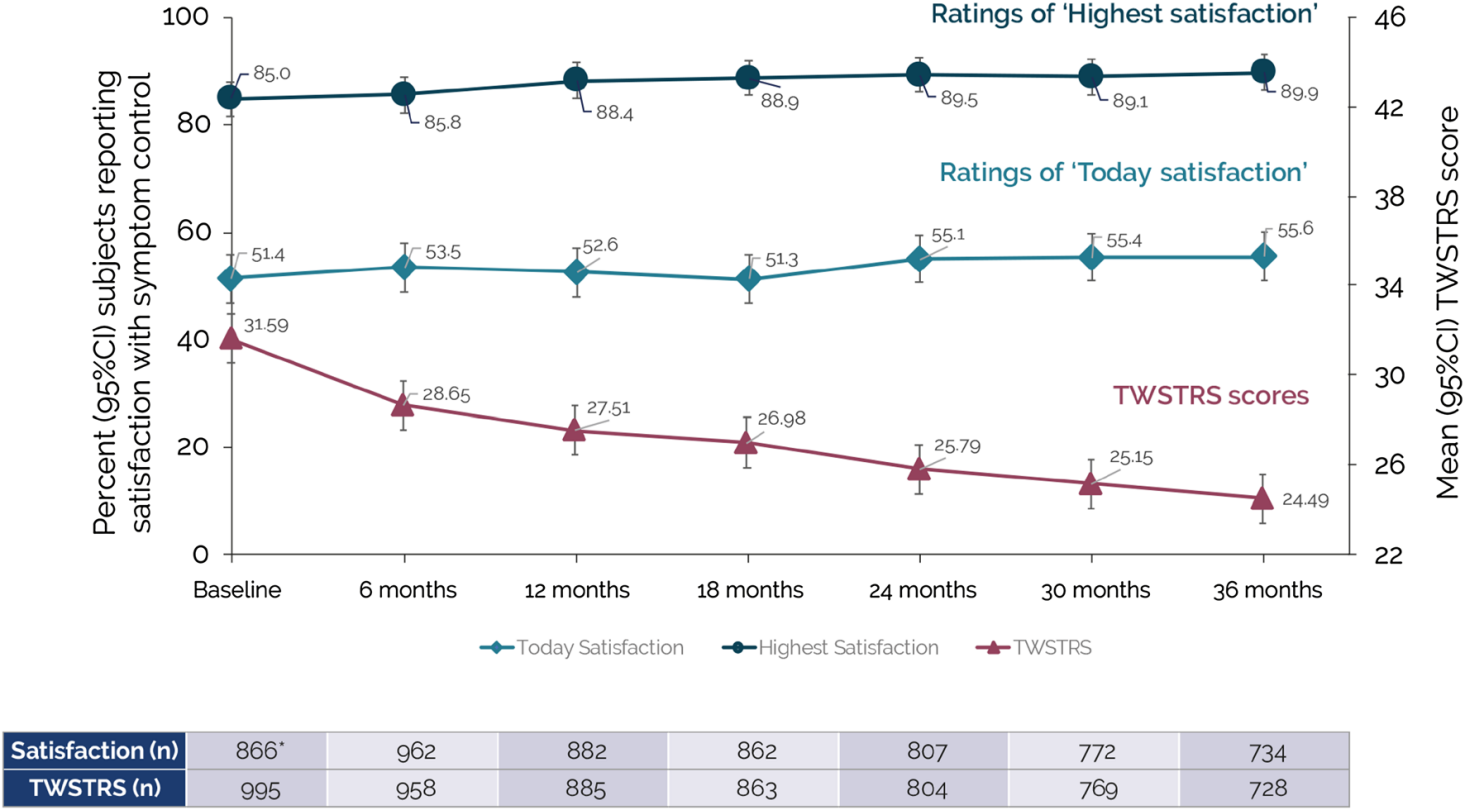
Subject satisfaction with symptom control and TWSTRS Total scores over 3 years. Baseline satisfaction was only assessed in patients who had previously been treated with botulinum toxin prior to study entry. *CI* confidence interval, *TWSTRS* Toronto Western Spasmodic Rating Scale

Overall, 55.6% of subjects reported satisfaction at the end of their last treatment cycle at 3 years (‘Today satisfaction’). Rates of satisfaction at peak effect (‘Highest satisfaction’) were higher, with 89.9% of subjects reporting satisfaction over the treatment cycle prior to the 3-year visit. In general, subject levels of satisfaction (both types) remained relatively stable from the first to the last injection visits, with ratings of ‘Highest satisfaction’ being consistently higher than ratings of ‘Today satisfaction’ (Fig. 2).

### Factors predictive of patient satisfaction

Results for the Step 1 univariate analysis (including all baseline factors tested) and Step 2 are presented in the Supplementary Appendix (Table e1 and e2). Multivariate analyses revealed that the Mean injection interval prior to satisfaction measurement was the only significant factor in the multivariate analyses associated with ‘Today satisfaction’ and was also associated with ‘Highest satisfaction’, where longer intervals were associated with higher satisfaction with symptom control (Table 3). Higher TWSTRS baseline total scores significantly predicted lower ‘Today satisfaction’.

**Table 3:** Factors associated with satisfaction in Step 3 (multivariate analysis of main study population)

Factor	Effect	*p* value (Wald Chi-square)	Odds ratio
Highest satisfaction			
Mean injection interval (weeks) prior to satisfaction measurement	Continuous	*p* = 0.0364	1.073 [1.010, 1.150]
Today satisfaction			
TWSTRS Total score	Continuous 0–85	*p* < 0.001	0.980 [0.970, 0.989]
Mean injection interval (weeks) prior to satisfaction measurement	Continuous	*p* = 0.0422	1.033 [1.003, 1.069]

*TWSTRS* Toronto Western Spasmodic Torticollis Rating Scale

### Clinical assessments and safety

Over the course of the study, no relevant changes were observed in the overall population in terms of the anatomic location of CD nor the predominant or secondary head/neck deviation patterns or components. However, we did observe some changes in those patients who were BoNT naïve at study entry. For this subgroup of patients, the proportion of subjects with simple CD (one pattern) increased from 27.9% at baseline to 32.2% at 36 months, and conversely the proportion of subjects with complex CD (at least two patterns) reduced from 72.1% to 66.1%. Taken overall, 10.2% of previously BoNT naïve subjects had a shift from rotation to laterocollis and 6.8% had a shift from laterocollis to rotation.

Mean TWSTRS Total scores (assessed at the end of each injection cycle) appeared to continually decrease over the course of the study (from 31.59 at baseline to 24.49 at 3 years) (Fig. 2). The mean ± SD reduction from baseline in TWSTRS Total score was − 6.97 ± 11.56 points at 3 years. There was a general shift of TWSTRS Severity scores from severe (TWSTRS severity score ≥ 15) to mild–moderate (TWSTRS severity score < 15). At 6 months, 15.8% subjects shifted categories from severe to mild–moderate and 7.4% shifted from mild–moderate to severe versus baseline. At 36 months, 26.1% subjects shifted from severe to mild–moderate and 5.5% shifted from mild–moderate to severe versus baseline. Likewise, CD-related tremor also tended to decrease as evidenced by subtle improvements in the Tsui categories of severity and duration. At 6 months, 12.5% subjects had improved tremor severity from baseline (a shift from severe to mild/none or from mild to none), while 5.7% subjects showed a worsening (from mild to severe or from none to mild/severe). At 36 months, 17.5% subjects had improved tremor severity from baseline and 10.0% had worsened. Improvement was also seen in Tsui duration categories. Subjects with continuous tremor decreased from 28.0% at baseline to 22.9% at 6 months and then remained roughly stable (22.5% at 36 months). Taken overall, the percent of subjects with a Tsui tremor component score of 4 (i.e., maximal severity and duration) reduced from 12.4% at baseline to 8.9% at 6 months, and then remaining relatively stable thereafter (score of 8.1% at 3 years).

Overall, the AE data reported were consistent with the known safety profile for aboBoNT-A and were as expected in this study population. The study did not raise any new safety concerns or changes to the known safety profile of aboBoNT-A in this indication.

## Discussion

The INTEREST IN CD2 study represents the largest CD patient cohort treated with BoNT-A to be followed longitudinally over 3 years. A previous single-cycle study, INTEREST IN CD1 [26], had shown that patient satisfaction with treatment is a valuable measure of treatment efficacy, and it was thus used as the primary outcome measure in this larger study. We show that, despite continued improvements in clinical features over 3 years, subject satisfaction with CD symptom control remains relatively constant.

In this study, nine in ten patients (89.9%) were satisfied with their symptom control at peak BoNT-A effect and just over half (55.6%) were still satisfied at the end of their injection cycle. These rates of satisfaction are similar to those reported in the survey conducted by Sethi et al. where 88.3% of patients reported satisfaction at peak effect and 60.8% were somewhat/very satisfied just prior to their next injection [26]. The lower level of satisfaction with symptom control on the day of the clinic visit is to be expected because (in most cases) at least 12 weeks had passed since the last injection when the therapeutic effects of the last injection are expected to be waning. In this context, it could even be suggested that the level of ‘Today’ satisfaction is rather high. Indeed, at the start of the study, we questioned whether subjects at some of the earliest recruiting centres (Cycle 1) understood the difference between the two types of satisfaction being assessed and provided extra training to those sites and all new recruiting sites. It is therefore reassuring that rates of ‘Today’ satisfaction remained relatively stable throughout the study. The reasons why patients are still satisfied at the end of a routine treatment cycle merit further investigation. It could be that these patients were benefiting from a long-lasting effect of BoNT treatment, which provided coverage of their symptoms over the treatment cycle. However, patients with long-term conditions often develop excellent therapeutic relations with their treating physician, and a good relationship is associated with better subjective outcomes [27], including greater treatment satisfaction [28, 29].

We found that those subjects with longer treatment intervals are significantly more likely to be satisfied with control of their symptoms than those with shorter intervals, both at peak effect and end of cycle. In terms of satisfaction at peak effect, it should be noted that many subjects were treated at centres which allowed for flexible/semi-flexible injection timings, and it may be those subjects who responded best and were able to achieve good symptom coverage over the full treatment cycle were the ones who had longer injection cycles. The reasons for greater satisfaction at end of cycle are harder to explain, as intuitively one could expect that a longer interval between injections would be associated with lower ‘Today’ satisfaction. Again, it could be that subjects with the best response were the ones who could manage longer injection cycles. Nevertheless, the observation that longer injection intervals do not negatively affect patient satisfaction with symptom control is of practical interest because longer intervals would help reduce the social (fewer appointments and injections) and economic (reduced drug costs, less physician time) burdens associated with the ongoing management of CD. Our results also confirm the clinical experience that patients with more severe CD (as evidenced by higher TWSTRS Total scores in this study) are less likely to be satisfied with their symptom control from established BoNT-A treatment at the end of cycle. This indicates a need for clinicians to help set clear and realistic expectations for therapy. Patient surveys have highlighted the high expectations patients have for their treatment, with over 60% of patients expecting freedom from spasms and/or freedom from pain and over half expecting to be able to return to a normal routine [7].

Our findings of relatively constant levels of satisfaction despite gradually improving disease severity (as evidenced by objective rating scales), indicate that factors other than symptom control also play a role in self-reported satisfaction. For example, most patients with CD also suffer from non-motor symptoms which may not respond to BoNT-A treatment and which may be associated with dissatisfaction [30]. According to the recent analysis of the minimally clinically important TWSTRS change reported by Espay et al. [31], the reduction of 7 points in Total-TWSTRS scores over 3 years represents a clinically meaningful improvement—despite the fact that these TWSTRS assessments were performed at the end of each treatment cycle. As such, these data are in line with the suggestion that patients do not allow their symptoms to return to baseline before requesting their next treatment. Indeed, the majority of sites who responded to the in-study survey reported some level of flexibility built into their service provision. A patient observed ‘BoNT waning of effect’ may actually represent the start of diminishing benefit and not a complete loss of benefit. Our observation may also reflect a potential disease modifying effect from repetitive injections. For example, Skogseid et al. reported on five patients who had discontinued from their long-term (≥1.5 years) BoNT treatment because ‘their symptoms had reduced to the extent that no further treatment was required’ [15].

The observation that changes in clinical presentation (i.e., patterns of head/neck deviation) were apparent in the subgroup of subjects who were previously naïve to treatment is of some practical importance. In routine practice, and particularly in busy BoNT injection clinics, time limitations often restrict the ability to regularly perform the comprehensive assessments as used in this study. Patients are often reinjected according to their prior injection schema. However, it is thought that a common cause of non-response to BoNT treatment is poor muscle choice (or other injection parameters) [32], and our data suggest that injectors should pay close attention to new patients in case their pattern of dystonia changes.

Key strengths of this study include its size, international reach and inclusion of all BoNT-A products. Many other studies have been restricted to one product and often one country [25–27]. Nevertheless, the fact that the majority of patients used aboBoNT-A in this study indicates some site selection bias. Indeed, many of the sites involved in the INTEREST IN CD1 study continued within the clinical study program. However, it should be noted that half (50.5%) of sites used multiple BoNT-A brands (mostly offering aboBoNT-A and onaBoNT-A). The lack of routine safety assessment is another limitation; however, the safety data collected for the sponsor’s own product are in line with the well-established literature [9, 12], and no new concerns were noted. Other limitations include those inherent to observational studies. For example, the level of missing data.

In summary, the results from this study provide a comprehensive overview of patient presentation during the routine, long-term management of CD with BoNT-A. While there was a clear tendency to reduce disease severity, the majority of patients required repeat injections to maintain therapeutic efficacy and satisfaction with symptom control. A good understanding the various factors associated with treatment satisfaction is vital when discussing the goals of treatment with patients (i.e., setting of realistic expectations) and for planning treatment regimens.

## Electronic supplementary material

Below is the link to the electronic supplementary material.

Supplementary file1 (PDF 168 kb)
