# Correction to: How satisfied are cervical dystonia patients after 3 years of botulinum toxin type A treatment? Results from a prospective, long-term observational study

**DOI:** 10.1007/s00415-019-09549-w

**Published:** 2019-10-09

**Authors:** Carlo Colosimo, David Charles, Vijay P. Misra, Pascal Maisonobe, Savary Om, the INTEREST IN CD2 study group, A. Abdulnayef, N. U. Adatepe, M. A. Araujo Leite, S. Badarny, O. Bajenaru, M. Bares, P. Bejjani, B. Bergmans, R. Bhidayasiri, H. Bozic, F. E. Cardoso Costa, C. Carlstrom, G. Castelnovo, M. H. Chang, Y. Y. Chang, T. M. Chung, M. Coletti-Moja, V. Delvaux, P. Dioszhegy, O. Dogu, W. Duzynski, E. Ehler, L. Espinosa Sierra, G. Fabbrini, J. Ferreira, A. Ferreira Valadas, C. Foresti, P. Girlanda, K. J. Goh, A. Graca Velon, S. Grill, T. Gurevitch, M. Hadidi, M. A. Hamimed, A. Hamri, T. Harrower, S. Hassin, P. Hedera, J. F. J. G. Hernandez, J. Hernandez Franco, B. Ho, S. L. Ho, A. Hughes, T. Ilic, J. S. Inshasi, C. W. Ip, S. Jamieson, R. D. G. Jamora, R. Jech, B. S. Jeon, A. Kaminska, M. Karpova, D. Khasanova, J. M. Kim, J. W. Kim, C. Y. Kok, A. Korenko, J. Korv, S. Koussa, T. Kovacs, A. Kreisler, P. Krystkowiak, W. Kumthornthip, C. H. Lin, F. Lundin, G. Lus, M. Magalhaes, A. N. Masmoudi, R. Mercelis, A. Misbahuddin, C. Moebius, B. Mohammadi, B. Nazem, K. Ng, G. Nurlu, J. Nyberg, D. Nyholm, S. Ochudlo, P. Otruba, R. Pfister, Z. Pirtosek, D. Pokhabov, S. Quinones Aguilar, G. Quinones Canales, S. Raghev, H. Rickmann, M. Romano, R. L. Rosales, I. Rubanovits, V. Santilli, L. Schoels, M. Simonetta-Moreau, M. A. Simu, Y. H. Sohn, S. Soulayrol, I. Supe, M. Svetel, T. Sycha, E. K. Tan, S. Timerbaeva, A. B. Tokcaer, R. Trosch, V. Tugnoli, V. Tumas, C. Van der Linden, A. Vetra, C. Vial, E. Vidry, D. Williams, S. Wimalaratna, C. Yiannikas

**Affiliations:** 1 Department of Neurology, Santa Maria University Hospital, Viale Tristano di Joannuccio 1, 05100 Terni, Italy; 2 Department of Neurology, Vanderbilt University Medical Center, Nashville, TN USA; 3 Department of Neurology, Imperial College Healthcare NHS Trust, London, UK; 4 Ipsen Pharma, Boulogne-Billancourt, France

## Correction to: Journal of Neurology 10.1007/s00415-019-09527-2

The article How satisfied are cervical dystonia patients after 3 years of botulinum toxin type A treatment? Results from a prospective, long‑term observational study, written by Carlo Colosimo, David Charles, Vijay P. Misra, Pascal Maisonobe, Savary Om on behalf of the INTEREST IN CD2 study group, was originally published electronically on the publisher’s internet portal (currently SpringerLink) on 09 Septembr 2019 without open access. With the author(s)’ decision to opt for Open Choice the copyright of the article changed on 13 September 2019 to © The Author(s) 2019 and the article is forthwith distributed under the terms of the Creative Commons Attribution 4.0 International License (http://creativecommons.org/licenses/by/4.0/), which permits use, duplication, adaptation, distribution and reproduction in any medium or format, as long as you give appropriate credit original author(s) and the source, provide a link to the Creative Commons licence and indicate if changes were made.

The original article has been corrected.

