# Supplementary material for: How satisfied are cervical dystonia patients after 3 years of botulinum toxin type A treatment? Results from a prospective, long-term observational study
Source: J Neurol. 2019 Sep 9;266(12):3038–46. doi: 10.1007/s00415-019-09527-2 (PMC6851034; doi:10.1007/s00415-019-09527-2)
Supplement: Supplementary file 1 — Supplementary file1 (PDF 168 kb) [file 415_2019_9527_MOESM1_ESM.pdf]

Supplementary File

Table e1. Step 1: Factors potentially associated with 'Today' and 'Highest' Satisfaction

Factor	Effect	Today Satisfaction		Highest Satisfaction	
		Odds Ratio (95% CI)	p-value	Odds Ratio (95% CI)	p-value
BMI Category (kg/m²)	25.0-29.9	0.905 (0.681, 1.204)	0.8621	1.342 (0.848, 2.163)	0.0658
	<18.5	0.804 (0.406, 1.592)		0.415 (0.192, 0.975)	
	>=30.0	0.965 (0.655, 1.424)		0.876 (0.505, 1.585)	
Age Category	18-30	1.046 (0.530, 2.097)	0.4998	0.796 (0.322, 2.266)	0.9921
	31-40	1.107 (0.692, 1.779)		1.113 (0.548, 2.377)	
	51-60	0.802 (0.559, 1.148)		1.039 (0.596, 1.794)	
	61-70	0.883 (0.608, 1.279)		1.074 (0.606, 1.899)	
	>70	1.168 (0.740, 1.852)		0.965 (0.495, 1.943)	
Sex	Female	0.972 (0.743, 1.271)	0.8377	0.901 (0.589, 1.355)	0.6218
First injection of BoNT for CD at baseline	Yes	0.755 (0.508, 1.121)	0.1630	0.944 (0.536, 1.781)	0.8505
Mean Injection Interval (weeks) prior to Satisfaction measurement	Continuous weeks	1.034 (1.003, 1.070)	0.0291	1.073 (1.010, 1.150)	0.0198

Factor	Effect	Today Satisfaction		Highest Satisfaction	
		**Odds Ratio (95% CI)**	**p-value**	**Odds Ratio (95% CI)**	**p-value**
Number of injection intervals prior to Satisfaction measurement	Continuous count	0.983 (0.945, 1.021)	0.3706	1.083 (1.025, 1.143)	0.0051
Other head/neck component at baseline	Complex deviation type	0.786 (0.582, 1.057)	0.1115	1.269 (0.815, 1.935)	0.2852
TWSTRS Total Score at baseline	Continuous 0-85	0.978 (0.968, 0.988)	<.0001	0.985 (0.971, 1.000)	0.0428
TWSTRS Pain Score at baseline	Continuous 0-20	0.971 (0.946, 0.996)	0.0256	0.937 (0.901, 0.974)	0.0010
Tremor at baseline	Yes	0.964 (0.751, 1.238)	0.7743	1.152 (0.786, 1.694)	0.4671
Jerk at baseline	Yes	0.952 (0.623, 1.460)	0.8210	1.810 (0.875, 4.391)	0.1154
Shoulder elevation at baseline	Yes	0.777 (0.605, 0.998)	0.0485	1.144 (0.781, 1.679)	0.4901
History of CD	Familial	0.962 (0.577, 1.613)	0.8814	1.324 (0.603, 3.496)	0.5101
Duration of CD (years)	<1	0.695 (0.418, 1.156)	0.1275	0.671 (0.335, 1.443)	0.5241
	>5	0.779 (0.596, 1.017)		0.851 (0.557, 1.284)	
Use of any injection guidance technique prior to satisfaction assessment	Yes	0.955 (0.742, 1.229)	0.7221	0.997 (0.679, 1.470)	0.9861
Focal cervical dystonia at baseline	Yes	1.272 (0.854, 1.896)	0.2368	1.084 (0.575, 1.911)	0.7917
Segmental dystonia at baseline	Yes	0.858 (0.534, 1.382)	0.5274	1.601 (0.735, 4.207)	0.2540

Factor	Effect	Today Satisfaction		Highest Satisfaction	
		Odds Ratio (95% CI)	p-value	Odds Ratio (95% CI)	p-value
Hemi dystonia at baseline	Yes	>999.999 (0.141, .)	0.2731	>999.999 (0.023, .)	0.6121
Multifocal dystonia at baseline	Yes	0.819 (0.299, 2.245)	0.6935	0.588 (0.186, 2.592)	0.4380
Generalised dystonia at baseline	Yes	0.517 (0.189, 1.324)	0.1698	0.347 (0.128, 1.097)	0.0692
BoNT injection for other indication within 12 months from baseline?	Yes	0.626 (0.265, 1.436)	0.2679	1.449 (0.418, 9.140)	0.6004
Tsui score at baseline	1	1.013 (0.750, 1.370)	0.9868	1.076 (0.687, 1.703)	0.7914
	2	1.046 (0.731, 1.501)		1.276 (0.737, 2.305)	
	4	1.070 (0.713, 1.610)		1.270 (0.687, 2.511)	
Total BoNT-A injection dose category based on last dose prior to satisfaction measurement	<Q1	0.957 (0.690, 1.331)	0.0564	0.746 (0.454, 1.257)	0.1193
	>Q3	0.686 (0.501, 0.938)		0.617 (0.389, 0.992)	
Surgical history for CD	Yes	0.521 (0.215, 1.199)	0.1255	0.483 (0.189, 1.485)	0.1868

p values are for the likelihood Chi-Square. Grey rows indicate entry into Step 2.

BMI: body mass index; BoNT: botulinum neurotoxin; BoNT-A: botulinum neurotoxin type A; CD: cervical dystonia; CI: confidence interval; Kg: kilogram; m^2: meter square; Q: quartile; TWSTRS: Toronto Western Spasmodic Torticollis Rating Scale

Table e2. Step 2: Test of independence for factors identified in Step 1

Interaction	Test of independence[1]	p-value	Decision
**Today satisfaction**			
**Interaction**	**Test of independence[1]**	**p-value**	**Decision**
**Today satisfaction**			
Baseline TWSTRS pain * Baseline TWSTRS total	Pearson correlation	<.0001	baseline TWSTRS pain dropped
Baseline TWSTRS pain * Mean Injection Interval	Pearson correlation	0.8703	
Baseline TWSTRS * mean Injection Interval	Pearson correlation	0.8205	
Baseline TWSTRS pain * BoNT-A injection dose category	Spearman correlation	0.0029	
Baseline TWSTRS pain * Duration of CD	Spearman correlation	0.2570	
Baseline TWSTRS total * BoNT-A injection dose category	Spearman correlation	<.0001	BoNT-A injection dose category dropped
Baseline TWSTRS total * Duration of CD	Spearman correlation	0.2430	
mean Injection Interval * BoNT-A injection dose category	Spearman correlation	0.0442	
mean Injection Interval * Duration of CD	Spearman correlation	0.0262	
Baseline shoulder elevation * Baseline generalised CD	Chi-Square	0.0623	
Baseline shoulder elevation * BoNT-A injection dose category	Chi-Square	0.0105	
Baseline shoulder elevation * Surgical history	Chi-Square	0.0029	
Baseline shoulder elevation * Duration of CD	Chi-Square	0.7401	
Baseline generalised CD * BoNT-A injection dose category	Chi-Square	0.2313	

Baseline generalised CD * Surgical history	Chi-Square	0.3553	
Baseline head/neck component * Baseline shoulder elevation	Chi-Square	<.0001	Baseline head/neck component dropped
Baseline head/neck component * Baseline generalised CD	Chi-Square	0.0175	
Baseline head/neck component * BoNT-A injection dose category	Chi-Square	0.0619	
Baseline head/neck component * Surgical history	Chi-Square	0.2674	
Baseline head/neck component * Duration of CD	Chi-Square	0.8779	
BoNT-A injection dose category * Surgical history	Chi-Square	0.7309	
NAIVE * Baseline shoulder elevation	Chi-Square	0.1072	
NAIVE * Baseline generalised CD	Chi-Square	0.9952	
NAIVE * Baseline head/neck component	Chi-Square	0.4545	
NAIVE * BoNT-A injection dose category	Chi-Square	0.0063	
NAIVE * Surgical history	Chi-Square	0.2940	
NAIVE * Duration of CD	Chi-Square	<.0001	NAIVE dropped
Duration of CD * Baseline generalised CD	Chi-Square	0.0604	
Duration of CD * BoNT-A injection dose category	Chi-Square	0.0008	BoNT-A injection dose category dropped
Duration of CD * Surgical history	Chi-Square	0.0799	
Baseline TWSTRS pain * Baseline shoulder elevation	Pooled t-test	<.0001	Baseline shoulder elevation dropped
Baseline TWSTRS pain * Baseline generalised CD	Pooled t-test	0.2446	
Baseline TWSTRS pain * Baseline head/neck component	Pooled t-test	0.0065	
Baseline TWSTRS pain * NAIVE	Pooled t-test	0.0365	

Baseline TWSTRS pain * surgical history	Pooled t-test	0.0200	
Baseline TWSTRS total * Baseline shoulder elevation	Pooled t-test	<.0001	Baseline shoulder elevation dropped
Baseline TWSTRS total * Baseline generalised CD	Pooled t-test	0.0036	
Baseline TWSTRS total * Baseline head/neck component	Pooled t-test	<.0001	Baseline head/neck component dropped
Baseline TWSTRS total * NAIVE	Pooled t-test	0.0573	
Baseline TWSTRS total * Surgical history	Pooled t-test	0.0167	
mean Injection Interval * Baseline shoulder elevation	Pooled t-test	0.5303	
mean Injection Interval * Baseline generalised CD	Pooled t-test	0.0391	
Mean Injection Interval * Baseline head/neck component	Pooled t-test	0.8410	
**Highest satisfaction**			
**Interaction**	**Test of independence[1]**	**p-value**	**Decision**
Baseline TWSTRS pain * Baseline TWSTRS total	Pearson correlation	<.0001	Baseline TWSTRS pain dropped
Baseline TWSTRS pain * Mean injection interval	Pearson correlation	0.8703	
Baseline TWSTRS pain * Number of injection intervals	Pearson correlation	0.1748	
Baseline TWSTRS total * Mean injection interval	Pearson correlation	0.8205	
Baseline TWSTRS total * Number of injection intervals	Pearson correlation	0.9860	
Number of injection intervals * Mean injection interval	Pearson correlation	<.0001	Number of injection intervals dropped

Baseline TWSTRS Pain * BMI category	Spearman correlation	0.0100	
Baseline TWSTRS Pain * BoNT-A injection dose category	Spearman correlation	0.0029	
Baseline TWSTRS * BMI category	Spearman correlation	0.1144	
Baseline TWSTRS * BoNT-A injection dose category	Spearman correlation	<0.0001	
Mean injection intervals*BMI category	Spearman correlation	0.8142	
Mean injection intervals* BoNT-A injection dose category	Spearman correlation	0.0442	
Number of injection intervals*BMI category	Spearman correlation	0.8555	
Number of injection intervals* BoNT-A injection dose category	Spearman correlation	0.2047	
Baseline generalised CD* BoNT-A injection dose category	Chi-Square	0.2313	
Baseline generalised CD*Surgical history	Chi-Square	0.3553	
Jerk at baseline * Baseline generalised CD	Chi-Square	0.8222	
Jerk at baseline* BoNT-A injection dose category	Chi-Square	0.1497	
Jerk at baseline* Surgical history	Chi-Square	0.4181	
BMI category*Baseline generalized CD	Chi-Square	0.0006	BMI category dropped
BMI category*Jerk at baseline	Chi-Square	0.1648	
BMI category* BoNT-A injection dose category	Chi-Square	<0.0001	BMI category dropped
BMI category*Surgical history	Chi-Square	0.7384	
BoNT-A injection dose category*Surgical history	Chi-Square	0.7309	
Baseline TWSTRS Pain * Baseline generalized CD	Pooled t-test	0.2446	

Baseline TWSTRS Pain *Jerk at baseline	Pooled t-test	<0.0001	Jerk at baseline dropped
Baseline TWSTRS Pain * Surgical History	Pooled t-test	0.0200	
Baseline TWSTRS * Baseline generalized CD	Pooled t-test	0.0036	
Baseline TWSTRS * Jerk at baseline	Pooled t-test	<0.0001	Jerk at baseline dropped
Baseline TWSTRS *Surgical History	Pooled t-test	0.0167	
Mean Injection intervals * Baseline generalized CD	Pooled t-test	0.0391	
Mean Injection intervals * Jerk at baseline	Pooled t-test	0.8519	
Mean Injection intervals * Surgical History	Pooled t-test	0.6602	
